# Stability of the anterior arm of three different Hyrax hybrid expanders: an in vitro study

**DOI:** 10.1590/2177-6709.23.1.037-045.oar

**Published:** 2018

**Authors:** Gonzalo de la Iglesia, André Walter, Fernando de la Iglesia, Heinz Winsauer, Andreu Puigdollers

**Affiliations:** 1 ​​Universitat Internacional de Catalunya, Department of Orthodontics (Barcelona, Spain).; 2 Private practice (Barcelona, Spain).​

**Keywords:** Microscrew, Mini-implant, Hybrid hyrax expander, Rapid maxillary expansion, Abutment stability

## Abstract

**Introduction:**

The force applied to the teeth by fixed orthopaedic expanders has previously been studied, but not the force applied to the orthodontic mini-implant (OMI) used to expand the maxilla with Hyrax hybrid expanders (HHE).

**Objective:**

The aim of this article was to evaluate the clinical safety of the components (OMI, abutment and double wire arms) of three different force-transmitting systems (FTS) for conducting orthopaedic maxillary expansion: Jeil Medical & Tiger Dental™, Microdent™ and Ortholox™.

**Methods:**

For the realization of this *in vitro* study of the resistance to mechanical load, three different abutment types (bonded, screwed on, and coupling) and three different OMIs’ diameters (Jeil™ 2.5 mm, Microdent™ 1.6 mm and Ortholox™ 2.2 mm) were used. Ten tests for each of these three FTS were carried out in a static lateral load in artificial bone blocks (Sawbones™) by a Galdabini universal testing machine, then comparing its performance. Comparisons of loads, deformations and fractures were carried out by means of radiographs of FTS components in each case.

**Results:**

At 1- mm load and within the elastic deformation, FTS values ranged from 67 ± 13 N to 183 ± 48 N. Under great deformations, Jeil & Tiger™ was the one who withstood the greatest loads, with an average 378 ± 22 N; followed by Microdent™, with 201 ± 18 N, and Ortholox™, with 103 ± 10 N. At 3 mm load, the OMIs shaft bends and deforms when the diameter is smaller than 2.5 mm. The abutment fixation is crucial to transmit forces and moments.

**Conclusions:**

The present study shows the importance of a rigid design of the different components of HHEs, and also that HHEs would be suitable for maxillary expansion in adolescents and young adults, since its mean expansion forces exceed 120N. Furthermore, early abutment detachment or smaller mini-implants diameter would only be appropriate for children.

## RESUMO

**Introdução:**

a força aplicada sobre os dentes por expansores ortopédicos fixos já foi estudada antes, mas não a força aplicada sobre os mini-implantes ortodônticos (MIOs) usados para expandir a maxila com expansores do tipo Hyrax híbrido (EHH).

**Objetivo:**

o objetivo desse artigo foi avaliar a segurança clínica dos componentes (MIO, *abutment* de fixação, e braços de fio duplo) de três sistemas de transmissão de força (STF) usados para expansão ortopédica da maxila: Jeil Medical & Tiger Dental™, Microdent ™ e Ortholox ™.

**Métodos:**

para realizar esse estudo *in vitro* sobre a resistência à carga mecânica, foram usadas três tipos de sistema de fixação (colado, aparafusado e *coupling*) e MIOs de três diâmetros diferentes (Jeil™ 2,5 mm; Microdent™ 1,6 mm e Ortholox™ 2,2 mm), com suas respectivas mecânicas de STF. Foram realizados 10 testes para cada STF, usando uma carga lateral estática em blocos de osso artificial (Sawbones™), com uma máquina universal de testes e, depois, comparou-se, por meio de radiografias, os desempenhos, levando-se em consideração as cargas, deformações e fraturas dos componentes de cada STF.

**Resultados:**

com a carga a 1 mm e sem exceder o limite de deformação elástica, os valores dos STFs variaram de 67 ± 13 N a 183 ± 48 N. Sob deformações maiores, o sistema Jeil & Tiger™ foi o que apresentou maior resistência às cargas elevadas, com valor de 378 ± 22 N; seguido pelo Microdent™, com 201 ± 18 N, e Ortholox™, com 103 ± 10 N. Com a carga a 3 mm, o eixo do MIO se dobrou e deformou quando seu diâmetro era menor que 2,5 mm. O *abutment* de fixação é crucial para a transmissão das forças e momentos.

**Conclusões:**

o presente estudo evidenciou a importância da rigidez no *design* dos diferentes componentes dos STFs dos EHH. Também revelou que eles são adequados para a expansão da maxila em adolescentes e adultos jovens, pois as forças de expansão, em média, excederam os 120N. Além disso, a desconexão precoce do *abutment* ou o uso de mini-implantes de menor diâmetro no *design* do STF seriam apropriados apenas em crianças.

## INTRODUCTION

Maxillary expansion is the therapeutic procedure in which transverse dimension of the maxilla is augmented using different type of appliances. With fixed maxillary expanders, a maxillary expansion can be achieved, by separating the palatal suture.

Dr. Emerson C. Angell described this maxillary expansion treatment in 1860. He was the first to apply a screw in premolars area, therefore expanding the arch a quarter of an inch in two weeks, producing an interincisal diastema. He achieved bilateral expansion by mechanically forcing the midpalatal suture, in a clinical case of transverse deficiency.^1^

Since then, maxillary expansion has been performed with different dental appliances, using various expansion protocols. Those should be placed during the growth and development stage of the patient, given that bones have not yet been completely developed. They are used to promote rapid expansion of the maxilla, so it can grow properly, preventing from the occurrence of future problems, such as: narrow smile, asymmetry deviation of the mandible, crowding and other problems.^2^

**Figure 1:**
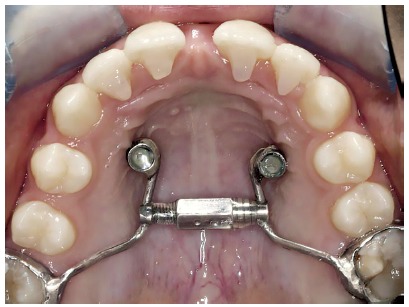
Hyrax hybrid expander (HHE) adapted to two orthodontic mini-implants (OMI).

There are several types of fixed orthopaedic expanders, and also their modified versions. The most used expanders are: Haas, Hyrax and McNamara. Recently, other types of expanders have been used,^3^ which are called “Hybrid Hyrax Expanders” (HHE) and consist of posterior arms attached to the molar bands (dental anchorage); anterior arms, attached to orthodontic mini-implants (OMI) (bone anchorage) via abutments or caps; and the expansion screw. The difference from the classic expanders is that the hybrid type features OMIs (Fig 1).

Triaca et al.^4^ (1992) and Wehrbein et al.^5^ (1996) were the pioneers in the introduction of palatal implants for the correction of Angle Class II. Their drawback was that they were osseointegrated, thus requiring a more complex removal than usual. Nowadays it does not happen, since the OMIs now used have no treated surface or titanium alloys Type V (biocompatible), being made of steel or lactic-glycolic acid (slowly biodegradable), preventing osseointegration.

Currently, rapid maxillary expansion (RME) can be performed in children by using HHE, which counts on the aid of the bone anchor of OMIs in the anterior palate and a dental anchor in the first upper molars. These tooth and bone-borne expanders are used mainly to gain space and to obtain a transversal increase of the maxilla, but other uses are also possible, such as space maintainers or for anchorage purposes.^6^ ^,^ ^7^

OMIs have the advantage of being anchored to the anterior palate; thus, disjunctions are purer, with more bone movements and less tooth movements.^6^

By activating the expansion screw of the HHE, the force is transmitted by its anterior arms to the respective abutments and OMIs inserted to the anterior palate, and by its posteriors arms to the molars. The forces applied to the teeth by fixed orthopaedic expanders have already been studied,^8^ and the disadvantage of molar tipping is described in the scientific literature.^9^ The major contribution of the present article is the *in vitro* simulation of the forces that OMIs will be submitted to when expansion is accomplished.

Thus, the aim of this article was to evaluate clinical safety of the components (OMI, abutment and double wire arms) of three different force-transmitting systems (Jeil Medical & Tiger Dental™, Microdent™ and Ortholox™) used to conduct orthopaedic maxillary disjunctions.

## MATERIAL AND METHODS

The three types of self-drilling OMIs analysed in this study are commercially available, being manufactured with a titanium alloy (Ti 6Al-4V ELI). 

**Figure 2:**
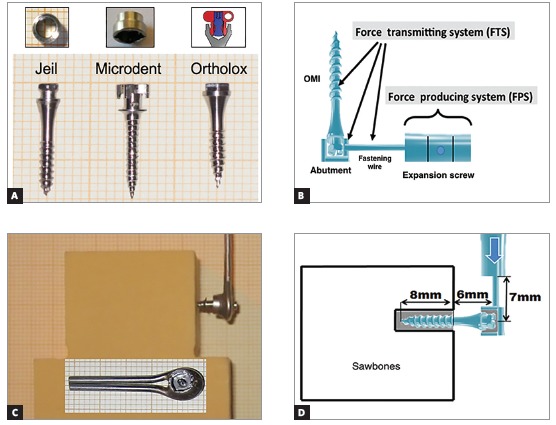
Illustration of the FTS.

The characteristics examined in each OMI and their variations are presented in Table 1 and Fig 2A. OMIs dimensions and abutment characteristics were obtained from the manufacturer’s specifications. 

**Table 1:** OMIs dimensions and abutment fixations types.

	Outer diameter	Inner diameter	Total length	Abutment type	Abutment fixation
Jeil & Tiger	2.5 mm	1.8 mm	14.5 mm	collar	Bonded / resin
Microdent	1.6 mm	1.1 mm	15.0 mm	cap	Screwed-on
Ortholox	2.2 mm	1.5 mm	14.0 mm	Push-button clipped cap	Snap-in coupling system

The Jeil & Tiger system (Jeil Medical, Seul, Korea & Tiger Dental, Bregenz, Austria) consists of a bonded/cemented collar over the OMIs head, with chemically-cured adhesive (Phase II, Reliance Orthodontic Products, Itasca, IL, USA). For the abutment removal, an extractor like a corkscrew is used and it allows the reuse of the OMI for other orthodontic objectives.^9^

The Microdent system (Microdent, Santa Eulalia de Ronçana, Barcelona, Spain) uses an internally fixated screw to join the abutment and the OMI. By unscrewing the inner screw, you can remove the abutment, making it possible to reuse the OMI for other orthodontic purposes.

The Ortholox system (Promedia Medizintechnik, Siegen, Germany) makes use of a coupling system. When the abutment is screwed, it widens, getting locked and clipped into the screw head, and this way fixing is achieved. 

As they are new systems, it was decided to analyse them by means of the present *in vitro* study. Their performance against a static lateral load exerted by a Galdabini machine (Cardano al Campo, Italy; Felben-Wellhausen, Switzerland) was compared. An universal servo-hydraulic Quasar 5 Galdabini machine (ISO 9001 certified company) was used to perform the tests, which is a desktop testing machine with a dual column for sample analysis (suitable for metals, plastics, composites and other materials), capable of exerting 100 kiloNewtons of load. We used Graphwork 5 (Galdabini, Cardano al Campo, Italy) software for programming and monitoring tests results, which allowed us to manage obtained information according to European, North American and International guidelines. This software allowed to manoeuvre the arm by remote control, and to perform the exact path desired. Load was measured in Newtons (N), whereas deformation was measured in micrometers (µm). 

The load arm of the Galdabini machine was used as the force producing system, simulating the expansion screw and transmitting the force to the components of the anterior arm of the HHE (force transmitting system, FTS) (Fig 2B). For this *in vitro* study on the resistance to mechanical load of the three FTS (Jeil Medical & Tiger Dental™, Microdent™ and Ortholox™), 10 mini-implants with its corresponding fixation abutment and double wire arms were used (Fig 2C). 

For the mechanical loading tests, artificial bone blocks (2 cm x 4 cm x 4 cm) were used (Sawbones™, density of 30 pcf) simulating the palate bone^10^ (Fig 2C). This selected material is a composite material for biomechanical tests, made of solid foam and used as alternative to human cadaver bone, used in other *in vitro* studies.^11^ ^,^ ^12^ Also, it presents many advantages, as: is a more reliable and standardized means for loading tests, and it doesn’t require preservation or handling requirements. For placing OMIs in the artificial bone blocks, a manual screwdriver was used to insert them leaving exposed only the OMI head, with its abutment, and the double wire fixation (Fig 2D). This OMIs placement was performed perpendicularly to the surface of the artificial bone block, at a 12- mm distance from the upper edge.

The bone blocks with OMIs, abutments and wire arms were fixed in the testing bench of the Galdabini machine, to prevent any movement or displacement. The vertical loading powerarm held on the wire arms of the FTS. A lateral loading was applied, transmitting force to the wire arms, abutment and OMI. These system simulated to the maximum the conditions of rapid maxillary expansion (RME). The movement of Galdabini machine’s powerarm was configured with a continuous push (arm movement) up to 6 mm, corresponding to a theoretical 12 -mm expansion in a human mouth (given that the expansion is performed bilaterally). 

The two steel fixation wires (Dentaurum, Ispingen, Germany), 1.5-mm diameter for each wire and 26-mm length (from the centre of the screw to the wire end), were laser-welded and joined to the abutment, for each of the OMIs (n = 30) of the three different FTS. The load was applied to the wires as shown in Figure 2D.

The distance from the surface of the artificial block to the double fixation wires measured 6 mm, representing the average thickness of the palatal gingiva (??#8197;4 mm) and the length of OMIs’ head with fixed abutment (2 mm)^13^ ^,^ ^14^ (Fig 2D). 

The distance from the end of the Galdabini machine arm to the OMIs shaft was 7 mm, reflecting the 7 -mm average length of the HHE anterior arm^6^ (Fig 2D). The Galdabini load arm moved at a 1 mm/30” speed, thus, gradually increasing the shear force and the momentum in the FTS.

**Figure 3:**
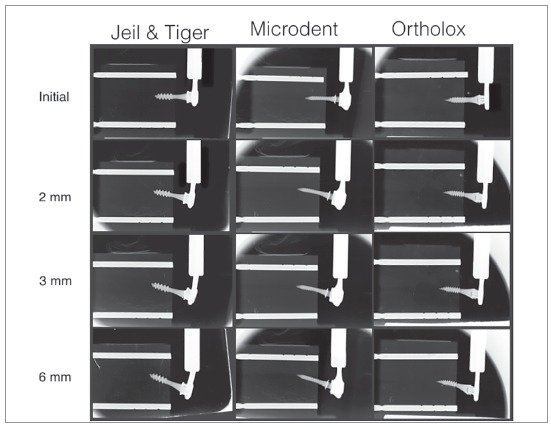
X-ray sequence (initial, 2 mm, 3 mm and 6 mm) of Jeil & Tiger, Microdent and Ortholox (left to the right) with double fastening wire.

For each test, radiographs were taken (Trophy^®^, Marne La Vallee, France) using occlusal films (5.7 x 7.6 cm): initial, at 2 mm, 3 mm and 6 mm of lateral load movement (Fig 3). The radiographs were performed at a perpendicular distance of 70 mm from the occlusal film to the wires, abutments and OMIs. Thus, an in-depth analysis of what had occurred (displacement, deformation or fracture) was carried out for each FTS, and deformation angles were measured using Adobe Photoshop CS6^®^software (Adobe Systems Inc., San Jose, California, USA).

**Figure 4:**
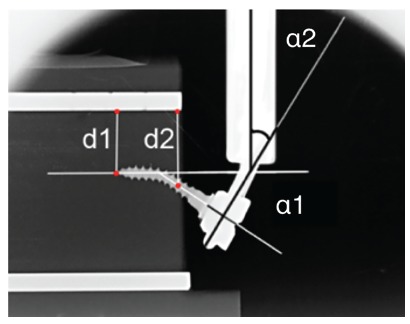
D1 and D2 distances; angle 1 and angle 2.

To help visualizing the deformation, two parallel radiopaque reference poles (fiduciary markers, 2-mm diameter) were placed in each artificial bone block (in the upper and lower sides). The upper marker identified a reference line (RL) used to measure deformation and displacement during charging. The lower marker served as an additional control of the image’s parallelism (Fig 4).

In Figure 4, D1 represents the distance between the OMI’s inserted tip and the RL; D2 is the distance between the OMIs axis and the RL measured from the surface of the bone block. The deformation of the pin or internal diameter was evaluated by measuring the α1 angle (angle between the original and final position of the axis of the OMIs) (head-neck-tip)), previously and subsequently to the load. The deformation of the wire arm or fixation wires (α2) was evaluated by measuring the angle between the vertical Galdabini arm and a line perpendicular to OMIs shaft (head-neck), at the wire insertion level (Fig 4). 

Descriptive statistics, with average and standard deviation (SD), were calculated for distances, angles and loading forces. Statistical analysis for general comparisons of the different obtained results for the three investigated trademarks were carried out by the Kruskal-Wallis test, with *p*< 0.05 being considered statistically significant. 

Comparisons between two trademarks were done with the *U* Mann-Whitney test, applying the Bonferroni correction, which is more reliable to analyse mechanical differences (significance level at *p* < 0.017) The statistical analysis was performed using SPSS v. 18.0 software (IBM Corp. New York, USA).

## RESULTS

**Figure 5:**
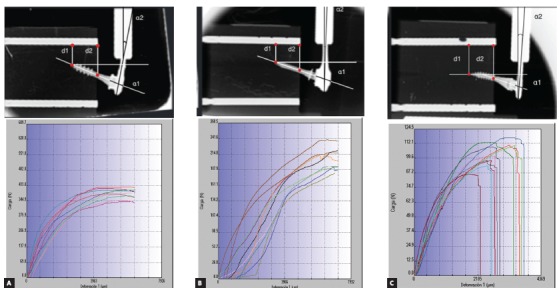
Tests performed for the three FTS: A) Jeil Medical & Tiger Dental, B) Microdent and C) Ortholox.

Results obtained in the tests (n = 30) made with the three different FTS are shown in Table 2 and Figure 5. The average loads at 1 mm, 3 mm and 6 mm were calculated for the 10 OMIs. The wire/OMIs deformations and displacements at 3 mm were calculated as well. 

**Table 2:** Loads, deformations and displacements of the 3 FTS.

					P values *	P values **	P values **	P values **
		J & T	Microdent	Ortholox	J & T vs. Md vs. Orthx	J & T vs. Md	J & T vs. Orthx	Md vs. Orthx
1-mm load (n=10)	Newton	183 ± 48	67 ± 13	76 ± 5	0	0	0	0.533
3-mm load (n=10)	Newton	323 ± 32	160 ± 14	109 ± 4	0	0	0.001	0.001
6-mm load (n=10)	Newton	378 ± 22	201 ± 18	102 ±10	0	0	0	0
Distance 1 (n=5)	mm	-1 ± 0.6	0 ± 0	-0.5 ± 0.4	0.014	0.008	1	0.032
Distance 2 (n=5)	mm	1.96 ± 0.2	1.56 ± 0.2	1.68 ± 0.1	0.013	0.016	0.032	0.31
α1 angle (n=5)	degrees	21 ± 1.6	15 ± 2.2	10 ± 1.8	0.002	0.008	0.008	0.016
α2 angle (n=5)	degrees	8.4 ± 1.7	4.2 ± 1.5	0 ± 0	0.002	0.011	0.005	0.005

J & T = Jeil Medical and Tiger Dental, Md = Microdent, Orthx = Ortholox.

* For Kruskal-Wallis test (J & T vs. Md vs. Othx), p < 0.05 was statistically significant.

** For Mann-Whitney U test with Bonferroni correction, p < 0.017 was statistically significant.

In the graphics shown in Figure 5, quantitative values in terms of load and deformation were plotted for each performed test, which are distinguished by different colours. As the load moved forward for 60 seconds, (vertical axis, in Newtons) deformation was measured (horizontal axis, in microns) for each FTS.

The behaviour observed in the J&T FTS (Fig 5A) was similar for the 10 studied OMIs, with more inclination of the OMI toward the direction of loading force and a clear deformation on the double fixation wires. This system withstood the greatest load, with an mean maximum value of 378 ± 22 N. This FTS supported a mean maximum deformation of 6340.8 µm without fracture (Table 2, Fig 5A). At 1-mm lateral load, the transmitted force was 183 ± 48 N; and at 3-mm load, the tip of the OMI moved 1 ± 0.6 mm in the opposite direction to the applied force. Shaft displacement and fixation wires deformation were the largest observed, being statistically significant when compared to the others FTS (*p* ranged from 0.008 to 0.011). 

The Microdent FTS (Fig 5B) showed to be capable of enduring the maximum deformation without fracturing (mean = 7138.8 µm). It also was the second of the three FTS that most resisted to load, obtaining an mean value of 201 ± 18 N (Table 2). The Microdent OMI presented an clear inclination toward the load direction. At 1-mm lateral load, this FTS was capable to transmit a mean load of 67 ± 13 N; and, at 3-mm load, OMI shaft and the abutment/head fixation bends and deforms without movement of OMI’s tip, resulting in less OMI displacement (Fig 3). The mechanical comparisons at 3 mm between Jeil & Tiger and the Microdent FTS system were statistically significant for all measurements (*p* < 0.001 to 0.016), although not significant between Microdent and Ortholox for the distances 1 and 2, due to similar OMIs displacement (*p*= 0.032 and 0.310, respectively).

In Figure 5C and Table 2, it is evident that the Ortholox FTS was the one with less load transmitting capacity, with a mean maximum load of 102 ± 10 N. At 1 mm, it transmitted a load of 76 ± 5 N, and at 3 mm the OMIs shaft showed less deformation when compared to Microdent FTS (10 ± 1.8^o^, *p*= 0.16) and greater abutment deformations. It was not capable to deform the fixation wires (0 ± 0^o^), thus resulting in a statistical significance when comparing the results to Jeil & Tiger and Microdent (*p*= 0.005, Table 2). A maximum deformation mean value of 3275.68 µm (Fig 5C) was obtained as result, causing an early unlatch of the abutment connection, and fracture at the abutment coupling fixation in the 10 samples submitted to load.

## DISCUSSION

Many factors influence the deformation of the OMIs such as bone density, place of insertion, the load applied, the retaining wire, the length and diameter of the OMI, among others.^15^ ^-^ ^18^ OMI’s diameters used in conventional hybrid expanders range from 1.8 to 2.0 mm.^6^ ^,^ ^9^ ^,^ ^19^ The present study analysed the mechanical behaviour under lateral load of three different OMIs with different diameters (1.6, 2.2 and 2.5 mm) and different abutment fixations, inserted at the same depth in artificial bone blocks, simulating the anterior arms of hybrid expanders. Although this is a limitation of the present study, it shows the importance of the design and stability of the three basic elements of the FTS (OMI, abutment and wires) and its mechanical interrelation with each other.

The use of artificial bone blocks to study the OMIs stability was also adopted in other studies.^11^ ^,^ ^12^ Lateral forces were applied and radiographs were performed to allow a better understanding of the mechanical behaviour. This setting simulated, as close as possible, the palatal bone and its properties, with a homogeneous bone density similar to that in adult patients^10^ (Fig 2C). Simulation of soft tissues was ignored, as they would barely influence the results. 

Present results show that the FTS with double fixation wires resisted maximal forces and moments ranging from 102 ± 10 N (Table 2) to 378 ± 22 N (Table 1). Nevertheless, great deformations were observed in the OMIs/double fastening wires, with values that are not suitable for clinical use (Fig 3). As for the abutment, there were cases in which it detached from the OMI and was not able to transmit forces and moments to the artificial bone (Fig 5C). It is known that after 1 mm of lateral load, the FTS underwent initial plastic deformation.^12^ Thus, under elastic deformation and for clinical uses, at 1 mm of load, the three FTS load values dramatically dropped, ranging from 67 ± 13 to 183 ± 48 N (Table 2).

The forces achieved in the 1-mm tests could be considered enough to perform the RME in children,^20^ ^,^ ^21^ but questionable for young adults, for the Microdent and Ortholox systems, both with similar load values (Table 2, *p*= 0.0533).^9^ Isaacson et al.^8^ recorded 100 N peak force for a 15.6-year-old girl. Sander et al.^22^ recorded, in 10 patients aged 9-13 years old, a maximum opening force of the maxillary suture of 120N. Boryor et al.^23^ recorded a force of 85N in a 73-year-old female corpse. As for the placement of the expander, the palatal mucosa was removed and it was placed directly into the maxillary bone, using four Forestadent OMIs (1,7-mm diameter and 8-mm length). With this data in mind, the 2.5-mm OMI can easily withstand these expansion forces and is more suitable for young adult patients, highlighting the importance of the larger diameter to support the expansion forces in these patients.^21^

The FTS used in this study created a moment when the lateral load was applied, due to the 4-mm thickness of mucosa in the anterior palate.^14^ Extraosseous component parts (double wire arm, abutment and part of OMI) acted as a lever, while the intraosseous part (OMI) resisted. When the lateral load was applied on the double wire arms, a moment was created, and therefore a deformation of the FTS.^24^ ^,^ ^25^ A cantilever was generated because the active portion (tip, turns and neck) of the OMI was inserted into the artificial bone and the other free end, not. The longer the lever arm, the higher load and deformation the FTS suffered.

The OMI has to be more rigid and preferably with a greater diameter to resist more deformation (α 1, *p*< 0.008,).^26^ Less deformations in the OMIs shaft or in the abutment fixation allows greater OMIs displacement and vice-versa.^26^ (Fig 5A, Table 2, distance 1 with tip movement and greater distance 2). 

The larger the inner diameter and alloy hardness, the OMI suffers less deformation and resists lateral load force better during expansion. This explains why the Microdent OMI deforms more than the other studied OMIs, as its diameter is 1.6 mm.

Walter et al.^13^ showed that OMIs characteristics as the internal diameter significantly affects the primary stability and the risk of fracture. Increasing the internal diameter by 0.1 mm notoriously improved the values of OMIs fracture by torsion.

Double fixation wires were used to ensure greater stability. Muchitsch et al.^27^ studied the rigidity of the double 1.5-mm diameter fixation wires, and concluded that the maximum force supported was 3.38 times higher in the double wires than in the single ones. They suggested that for use in adolescent patients, double welded wires positioned one above the other is what should be used. That disposition of the wires has proved successful in adolescents.^28^ The α2 angle demonstrated the importance of the OMI/abutment fixation stability, with higher deformation values in the fixation wires during load displacement, due to the higher forces and moments values (Table 2). On the other hand, a weak abutment fixation, detached by lower forces, does not allow the fixation wire deformation (Table 2, α2 angle, *p*= 0.005) and highlights the importance of abutment overlapping on the OMI’s head when facing greater lateral forces.^12^

In order to improve the result of HHE treatment, due to molar tipping risk caused by the dental anchor, Winsauer et al.^28^ introduced the MICRO-4 expander, a jaw expander that uses four OMIs as bone anchor.^29^ The advantage of this HHE design is that the force produced by the expansion screw is halved for each OMI, without risk of possible dental side effects. This HHE design was also modified to apply six OMIs as bone anchors (MICRO-6).^28^ It remains to be found if there are commercially available screws capable of producing 500 N of force or more.

The Jeil & Tiger FTS was the studied system that tolerated the highest force, up to 180 N, within the elastic deformation, but a question arises whether such force is needed or necessary and useful for maxillary expansion in adult patients. 

According to the clinical experience of the authors, the mean depth of insertion into the lateral anterior palate is of 3 to 5 mm.^29^ In order to improve bone anchor, OMI must be inserted to a greater depth, as there is enough bone thickness in that area.

## CONCLUSION

In conclusion, the present study showed that Jeil & Tiger and Microdent force transmitting systems (FTS) would be suitable for maxillary disjunction in adolescents, since its mean expansion forces exceed 120 N. Furthermore, the Ortholox FTS would not be appropriate, due to the unlatch of the abutment from the OMI, and because its mean force value did not exceed the required 120 N. The Jeil & Tiger FTS transmitted more force due to its 2.5 mm diameter.
